
JOURNAL INFORMATION
==============================
NLM Title Abbreviation: ScientificWorldJournal
Journal ID: ScientificWorldJournal
NLM Journal ID: 101131163
Journal ID: TSWJ

The Scientific World Journal

ISSN: 2356-6140
EISSN: 1537-744X
Publisher: Wiley

ARTICLE INFORMATION
==============================
PMCID: PMC6084262
PMID: 30147566
DOI: 10.1100/tsw.2001.211
Article ID: 572674
Article version: 1
Subjects: Short Report

Trophic Factor Withdrawal Induces a Novel Pathway: p38 MAPK Activates NHE1 Resulting in Intracellular Alkalinization, an Early Step in Apoptosis

Khaled Annette R. 1 *
Kim Kyungjae 1
Muegge Kathrin 2
Thompson Craig 3
Fliegel Larry 4
Durum Scott K. 1
Laboratory of Molecular Immunoregulation, National Cancer Institute, Frederick, MD, 21702, USA
SAIC, National Cancer Institute, Frederick, MD, 21702, USA
University of Pennsylvania, Philadelphia, PA, 19104, USA
University of Alberta, Edmonton, Alberta, Canada
*Annette R. Khaled: khaleda@ncifcrf.gov
Publication date: 2001
Electronic publication date: 2001 Dec 1
Volume: 1
First page: 93
Last page: 93
Copyright: Copyright © 2001 Annette R. Khaled et al.
Copyright year: 2001
License: This is an open access article distributed under the Creative Commons Attribution License, which permits unrestricted use, distribution, and reproduction in any medium, provided the original work is properly cited.
License URL: https://creativecommons.org/licenses/by/3.0/

==============================
Miami *Nature Biotechnology* Short Reports
*TheScientificWorld* (2001) 1 (S3), 93SR
ISSN 1532-2246; DOI 10.1100/tsw.2001.211

### TROPHIC FACTOR WITHDRAWAL INDUCES A NOVEL PATHWAY: P38 MAPK ACTIVATES NHE1 RESULTING IN INTRACELLULAR ALKALINIZATION, AN EARLY STEP IN APOPTOSIS

Annette R. Khaled[1*], Kyungjae Kim[1], Kathrin Muegge[2], Craig Thompson[3], Larry Fliegel[4], and Scott K. Durum[1]

[1]Laboratory of Molecular Immunoregulation, [2]SAIC , National Cancer Institute, Frederick, MD, 21702, [3]University of Pennsylvania, Philadelphia, PA, 19104, and [4]University of Alberta, Edmonton, Alberta, Canada
* khaleda@ncifcrf.gov

**INTRODUCTION.** The requirement for cytokines in hematopoiesis is partly attributable to a trophic activity, the protection of cells from apoptosis. For example, interleukin-7 (IL-7) protects lymphocyte progenitor cells from apoptotic death during T cell development (1,2). The survival effect of these cytokines has been partly attributed to the Bcl-2 family of proteins (2,3). However, the trophic action of cytokines involves more than the balance of Bcl-2 family members, since overexpression of Bcl-2 does not completely replace the cytokine survival signal (1,4). Hence, the death-inducing signaling pathways triggered early upon cytokine loss are yet to be fully defined.

**METHODS.** IL-3- and IL-7-dependent cell lines were evaluated following cytokine withdrawal for changes in cytosolic pH and mitochondrial biology by incorporation of fluorescent dyes, BCECF and JC-1. Cells were treated with pharmacological inhibitors of the sodium-hydrogen exchanger (NHE) and the Map Kinases (MAPK)s, p38 and ERK1/2. Protein levels and activities were assayed by Western blot and kinase assays. Mitochondrial function was determined through measurement of NADH and ADP/ATP levels. Death was evaluated by caspase activation and propidium iodide staining.

**RESULTS.** Loss of IL-7 or IL-3 in dependent cell lines induced the phosphorylation and activation of p38 MAPK, while the activities of other MAPKs (ERK and SAPK/JNK) declined. Phosphorylation of the downstream substrate of p38 MAPK, ATF-2, was observed in proportion to the increased kinase activity of p38 MAPK. Once activated, p38 MAPK mediated the direct phosphorylation of the pH regulator, the Na+/H+ exchanger 1 (NHE1), targeting specific residues in the C-terminus of NHE. Pharmacological inhibitors of p38 MAPK (but not ERK) prevented NHE phosphorylation in the absence of cytokines. As a result of NHE activation, a striking intracellular alkalinization occurred in which cytosolic pH rose steadily for 2-3 hours, peaking over pH 7.8 before returning to neutrality. This alkalinization could be reversed by addition of p38 MAPK inhibitors as well as NHE inhibitors. A critical outcome of alkalinization was the mitochondrial translocation of the pro-apoptotic protein, Bax (3). The pH rise triggered a conformational change in Bax, altering charged amino acids in the amino and carboxy- termini to expose hydrophobic membrane-seeking domains (3). Furthermore, alkalinization produced a transient hyperpolarization of the mitochondrial inner membrane, an effect blocked by oligomycin, inhibitor of the F0F1-ATPase. Functional consequences included depletion of ATP and the accumulation of NADH. Using cells from $Bax^{-/-}$ mice or overexpressing Bcl-2, we determined that cytosolic alkalinization and mitochondrial hyperpolarization are early events independent of the Bcl-2 family.

**DISCUSSION.** Our data suggest a model in which cytokine withdrawal rapidly activates p38 MAPK, leading directly to the phosphorylation of NHE1, the membrane ion exchanger responsible for intracellular alkalinization. This previously unrecognized pathway, activated early during apoptosis, results in the mitochondrial translocation of the pro-apoptotic protein Bax by a novel mechanism. The rise in cytosolic pH causes a conformational change in Bax (3). Further consequences include a transient rise in the mitochondrial membrane potential - an event that, together with Bax activation, produces severe metabolic stresses priming cells for death.

**REFERENCES.**

1. Hofmeister, R., Khaled, A.R., Benbernou, N., Rajnavolgyi, E., Muegge, K., and Durum, S.K. (1999) Cytokine Growth Factor Rev. 10, 41-60
2. Kim, K., Lee, C.K., Sayers, T.J., Muegge, K., and Durum, S.K. (1998) J. Immunol. 160, 5735-5741
3. Khaled, A.R., Kim, K., Hofmeister, R., Muegge, K., and Durum, S.K. (1999) Proc. Natl. Acad. Sci. U S A 96, 14476-14481
4. Nunez, B., London, L., Hockenbery, D., Alexander, M., McKearn, J.P., and Korsmeyer, S.J. (1990) J. Immunol. 144, 3602-3610
